# Three months of slackline training elicit only task-specific improvements in balance performance

**DOI:** 10.1371/journal.pone.0207542

**Published:** 2018-11-26

**Authors:** Louis-Solal Giboin, Markus Gruber, Andreas Kramer

**Affiliations:** Human Performance Research Centre, Sensorimotor Performance Lab, University of Konstanz, Universitätsstrasse, Konstanz, Germany; University of Florence, ITALY

## Abstract

Slackline training is a challenging and motivating type of balance training, with potential usefulness for fall prevention and balance rehabilitation. However, short-term slackline training seems to elicit mostly task-specific performance improvements, reducing its potential for general fall prevention programs. It was tested whether a longer duration slackline training (three months, 2 sessions per week) would induce a transfer to untrained tasks. Balance performance was tested pre and post slackline training on the slackline used during the training, on a slackline with different slack, and in 5 different non-trained static and dynamic balance tasks (N training = 12, N control = 14). After the training, the training group increased their performance more than the control group in both of the slackline tasks, i.e. walking on the slackline (time × group interaction with p < 0.001 for both tasks). However, no differences between groups were found for the 5 non-trained balance tasks, only a main effect of time for four of them. The long-term slackline training elicited large task-specific performance improvements but no transfer to other non-trained balance tasks. The extensive slackline training that clearly enhanced slackline performance did not improve the capability to keep balance in other tasks and thus cannot be recommended as a general fall prevention program. The significant test-retest effect seen in most of the tested tasks emphasizes the need of a control group to adequately interpret changes in performance following balance training.

## Introduction

Balance training seems to be an adequate intervention to enhance postural control and balance capability in athletes [1], and to prevent falls in older individuals [2]. However, the benefits acquired with balance training require sufficient doses of training and are not long lasting [2]. Therefore, to ensure adequate training adherence and training volume, high levels of enjoyment during the balance practice are necessary [3].

Recently, many studies focused on the outcomes of slackline training on balance performance [4–14]. Slackline training has a very large difficulty progression, a plethora of different movements to learn and is at the core of several sports (longlining, tricklining, etc.). Thus, slackline training can be considered as a particularly suitable type of balance training due to its motivating and challenging nature [15], which can bring higher levels of enjoyment and adherence than more traditional balance training (for example one leg stance on different unstable surfaces). However, a meta-analysis by Donath and colleagues focusing on the effects of 6 weeks or less of slackline training indicated that slackline training can induce large effects on the performance of slackline related tasks but only small to moderate effects on the performance of untrained tasks [15]. Slackline training seems to follow the balance training specificity principle [16, 17], i.e. short-term balance training seems to elicit a clear performance improvement in the tasks trained, but a very limited effect on non-trained tasks. A lack of large effects in untrained tasks could possibly reduce its suitability for general use, e.g. in fall prevention. However, the previously discussed meta-analysis is based on a limited number of studies and the task-specificity effect of slackline training remains to be more extensively studied.

It has been suggested that general balance training adaptations, i.e. improved performance in untrained tasks, could occur with longer training duration [15, 16]. Indeed, the neuromuscular adaptations induced by balance training are time dependant [1]. At the beginning of the training, the adaptations are mostly of neural origins, and are most probably very specific to the tasks trained [18]. Such effect could explain the task-specificity of short-term balance training and the lack of generalization [16]. A longer training duration may drive different adaptations at the neural and peripheral level than short-term training, and could induce generalization. Therefore, the aim of the present study was to test whether 12 weeks of slackline training could elicit general balance training adaptations in young healthy adults.

## Methods

### General overview

The effect of balance training on untrained static and dynamic tasks was measured. The extent of the balance training specificity principle was assessed by testing performance on a slackline with a different slack than the slackline used during the training. We hypothesized that the participants of the training group would improve more than the control group only in the slackline task, and that this improvement would be more pronounced for the slackline used during training.

### Participants

Twenty-eight young healthy participants were recruited among the students of the university. Two participants dropped out due to scheduling constraints (N = 26, 13 women, mean ± SD, 21.9 ± 2.3 years old, age range: 18–28 years, 174.2 ± 10;4 cm, 67.9 ± 7.8 kg). Participants were allocated in a control (N = 14, 8 women, 22 ± 3 years old, 175 ± 11 cm, 66 ± 8 kg) and a training group (N = 12, 5 women, 22 ± 2 years old, 174 ± 10 cm, 70 ± 7 kg). Participants were naïve from any slackline training and had no current or recent (less than a year) leg injury. Participants were told to not practice any form of balance training or change their usual physical activity during the whole length of the study (controlled with self-reported activity logs). Participants gave written informed consent before starting the study. The study was in accordance with the latest revision of the Declaration of Helsinki and specifically approved by the ethics committee of the University of Konstanz.

### Study design

The study was a controlled training study with baseline matched groups and pre-post training performance tests. After the pre training performance tests, participants were allocated to the training group or the control group. The allocation was done so performance in all the balance pre training tests was matched between the control and the training group. Further, we matched both groups according to their lower limb power. Indeed, it has been proposed that lower limb power may be a factor that can influence the learning of a balance task, and therefore should be taken into account when interpreting the effects of balance training [19]. For this, we used the minimization method by allocating the first 10 participants randomly to each group and then allocating the rest of the participant by matching variables of interest. This participant allocation method is considered to be a superior alternative to randomization [20, 21], especially in the case of a small sample size study like the present one. The training was followed by post training tests. There was at least 1 day of rest between the last training session and the post test. During training and testing the participants were barefoot.

### Training

The participants in the training group followed a supervised training during 12 weeks (2 sessions per week). The goal of the training was to teach participants to walk on a slackline. A training session always started with a 5–10 min warm-up consisting of mobilization exercises of the ankle, knee and hip joint. The warm-up was followed by four different exercises, all performed on the slackline. Each training session lasted around 45 min. There were at least 2 days of rest between 2 training sessions. Throughout the weeks, the difficulty of the exercises on the slackline was progressively increased. For example, trainees started to learn how to balance on one leg in the middle of the line first with help, and then without help from the supervisor. The next week, trainees had to do the same but with eyes closed. The next week, trainees had to walk along the line, starting from the middle of the line. When successful, trainees could do it but with eyes closed. The 12-week training was conducted over the winter term, with a 10-day break after the first six weeks (winter holidays break).

### Pre and post training tests

At the beginning of the pre- and post-tests, the participant’s weight and height was recorded. Then, the participant performed 8 different tests, in a counterbalanced order, with the same order pre and post training. The 8 different tests consisted of countermovement jumps (CMJ) and 7 different balance tasks. The participants performed 3 trials with 30 s of rest in between trials for the CMJ and 4 trials with 10 s of rest in between trials for each of the balance tasks. There was a rest of 1 min between tasks. The one-leg balance tasks (see below) were always performed with the preferred leg. The preferred leg was chosen by the subject after trying out a one leg stance on the floor with each leg.

#### CMJ

CMJs were performed with hands on the hips on a force plate (Leonardo Mechanograph GRFP, Novotec medical GmbH, Pforzheim, Germany). Prior to the first jump, participants were shown the correct jumping technique and were instructed to quickly drop to a squat and immediately jump as high as possible. From the force recordings, peak power relative to bodyweight (P_max_rel) was calculated.

#### Slackline trained

Participants started from a platform at one extremity of the slackline with their hands on the hips, and had to walk on the slackline for as many steps as possible. Between each step, the subject had to remain in a one leg stance position for 2 seconds. A go signal was given by the investigator before each step. Performance was determined as the number of steps performed before stepping or falling off the slackline. The task was performed on the slackline used during the three months of training (Slackline-Tools, width = 3 cm, height = 42 cm, length = 5 m, 14% height reduction with respect to the initial height when a 10 kg weight was placed in the middle of the line).

#### Slackline lab

The task was the same as described above. The only difference was that the task was performed on a different slackline with more slack, which was installed in our laboratory (GIBBON Slackline, width = 5 cm, height = 37 cm, length = 5 m, 25% height reduction when a 10 kg weight was placed in the middle of the line).

#### Sensoboard

The sensoboard task consisted of a one leg stance performed on a platform maintained over a hemisphere with elastics (SENSOBOARD Sensosport Gmbh). The participant, hand on the hips, had to step onto the tilt board from an elevated platform and maintain balance for as long as possible in a one leg stance (performance ceiling of 20 s). Performance was the time before losing balance, i.e. feet not touching the board or one side of the board in contact with the ground, measured with a stop watch.

#### Tilt board, medio-lateral (TBML)

The tilt board with medio-lateral axis of perturbation task was performed with hands on the hips and on one leg. The task consisted of bringing the platform of the tilt board into a horizontal position. One trial lasted 10 s. Performance consisted in the amount of time during which the platform was considered to be in equilibrium (i.e. when the platform was positioned in the horizontal plan ± 5 degrees). This was measured using a 12-camera motion capturing system (T40s, 200Hz, Vicon Nexus 1.8.5). Contrary to the sensoboard task, where the device was already in an equilibrium state at the beginning of the task, the TBML task requires a dynamic impulse to bring the tilt board into its “equilibrium” state. For more details, see [16].

#### Tilt board, antero-posterior (TBAP)

The tilt board with anteroposterior axis of perturbation task was identical to the TBML task except for the axis of perturbation. For more details, see [16].

#### One leg stance, eyes open (1LEO)

The 1LEO task consisted in maintaining a one leg stance with eyes open and hands on the hips on the force plate. Participants were instructed to stand as still as possible. Participants had a visual guide consisting of a mark placed at a height of 170cm on the opposite wall (4 m away). One trial lasted 10 s. Performance was defined as the ellipse area encompassing the centre of force trajectory recorded by the force plate.

#### One leg stance, eyes closed (1LEC)

The 1LEC task was identical to the 1LEO task, but performed with eyes closed.

### Analysis

P_max_rel was taken as the highest value obtained from the 3 CMJs performed. Power was calculated as the product of force and velocity (integral of acceleration) with the Leonardo GRFP 4.3 software. The same software was used to calculate the area of the ellipse containing the centre of force trajectory during the 1LEO and 1LEC tasks. Performance during the TBML and TBAP task was measured with motion capture system. The performance during each of the balance task was taken as the average performance of the 4 trials.

### Statistics

Statistics were done with JASP (JASP 0.8.3.1, University of Amsterdam) and R (3.5.1). Shapiro-Wilk tests showed non-normality in nearly all the data sets tested and Levene’s test showed heterogeneous variance at post level for the Slackline trained task and at pre level for the Slackline Lab task. Since ANOVAs are considered better than non-parametric tests even in case of assumption violation [22, 23], we still used two-way ANOVAs with repeated measures to test whether the changes in performance in slackline trained, slackline lab, sensoboard, TBML, TBAP, 1LEO and 1LEC tasks, as well as weight and P_max_rel differed between groups over time. In addition, a two-way ANOVA with repeated measures was used to assess differences in the training group’s improvement in the two slackline tasks, using task (slackline training and slackline lab) as the independent variable and time (pre and post) as repeated measure. We used the η^2^ to estimate the effect size of the interaction. As a control, we used non-parametric Aligned Rank Transform ANOVAs [24] to test changes in performance in slackline trained, slackline lab, sensoboard, TBML, TBAP, 1LEO and 1LEC tasks.

## Results

**Fig 1:**
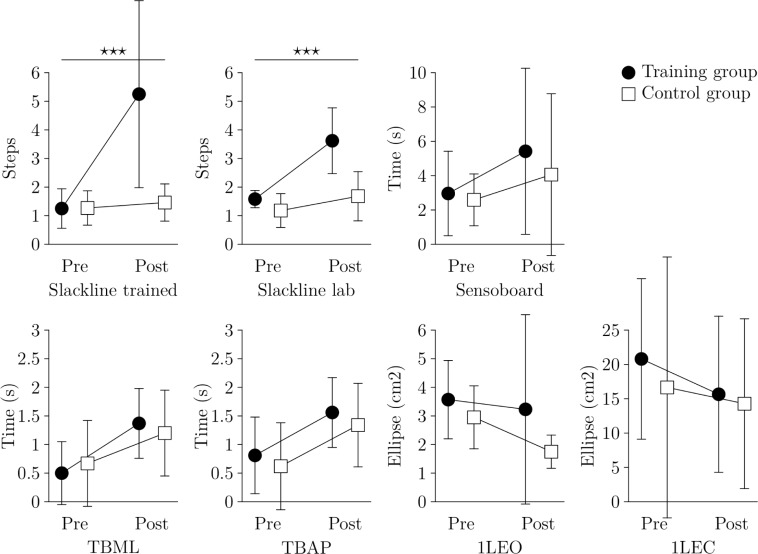
Balance task performance results. Performance for each balance task pre and post training for the training (black circle) and control group (white square). Error bars represent standard deviations. Horizontal bars with *** represent significant group × time interaction effects with a p-value < 0.001.

There was a significant time × group effect for both of the slackline tasks, see Fig 1 and Table 1. For the differences between the two slacklines, we observed only a time (F_1,11_ = 35.4, p < 0.001) but no task (F_1,11_ = 1.68, p = 0.22) or time × task effect (F_1,11_ = 3.32, p = 0.09).

**Table 1:** Statistical results. Results of the two-way ANOVAs with repeated measures for each of the seven tested balance tasks. The η^2^ estimates the effect size of the interaction. Abbreviations: TB tilt board, TBML tilt board medio-lateral, TBAP tilt board anteroposterior, 1LEO one-leg stance with eyes open, 1LEC one-leg stance with eyes closed.

Task	Time	Group	Time × group	η^2^
Slackline trained	F1,24 = 20.9; p < 0.001	F1,24 = 16.3; p < 0.001	F1,24 = 17.1; p < 0.001	0.28
Slackline lab	F1,24 = 41.8; p < 0.001	F1,24 = 24.2; p < 0.001	F1,24 = 15.4; p < 0.001	0.19
Sensoboard	F1,24 = 5.5; p = 0.028	F1,24 = 0.55; p = 0.46	F1,24 = 0.35; p = 0.56	0.012
TBML	F1,24 = 16.5; p < 0.001	F1,24 = 0.0006; p = 0.98	F1,24 = 0.96; p = 0.34	0.023
TBAP	F1,24 = 14.5; p < 0.001	F1,24 = 0.73; p = 0.4	F1,24 = 0.002; p = 0.96	0
1LEO	F1,24 = 27.5; p < 0.001	F1,24 = 3.3; p = 0.083	F1,24 = 0.06; p = 0.81	0.01
1LEC	F1,24 = 1.25; p = 0.27	F1,24 = 0.38; p = 0.54	F1,24 = 0.17; p = 0.68	0.07

For all other tested balance tasks we observed no group × time interaction effect, nor any main effects of group. A main effect of time could be observed for all the tasks except for the 1LEC task (see Table 1).

There was no difference of weight between groups pre or post training (Time: F_1,24_ = 4.1, p = 0.054; Group: F_1,24_ = 0.96, p = 0.34; Time × Group: F_1,24_ = 3.3, p = 0.08). There was also no difference of P_max_rel between groups or before and after the training (Time: F_1,24_ = 2.04, p = 0.16; Group: F_1,24_ = 2.14, p = 0.15; Time × Group: F_1,24_ = 0.24, p = 0.62; mean ± SD; control pre and post: 43.9 ± 6 and 44.4 ± 6 W/kg; training pre and post: 47.7 ± 9 and 48.6 ± 7 W/kg). The corresponding jump height were for the control group pre and post: 38.3 ± 6 and 40 ± 5 W/kg; and for the training group pre and post: 41.2 ± 8 and 44.9 ± 8 W/kg. The Aligned Rank Transform ANOVAs gave similar results as the parametric ANOVAs (not displayed).

## Discussion

After three months of slackline training, the training group significantly improved their performance in the two slackline tasks compared to the control group, but not in the untrained balance tasks.

In line with shorter training studies with a duration of few weeks [4–6, 9, 12, 25], slackline training induced only task-specific improvements in balance performance. This means that, in comparison to a control group, trainees were able to improve performance only for the task they trained. The lack of transfer effects clearly supports the balance training specificity principle and the concept that balance is not a general ability but more a sum of specific skills [16]. It must be noted that several slackline training studies reported transfer effects to some of the untrained tested balance tasks [8, 10, 11, 13]. However, transfer occurred only for a few of the tested tasks and therefore does not support the concept of an increased general balance capacity. Instead, it has been suggested that the transfer observed did not stem from a skill training per se, but rather neuromuscular adaptations [15, 16]. Indeed, balance training can improve the capacity to produce force in a limited amount of time [26], which, in theory, could lead to a faster capacity to counteract a posture perturbation. This could partly explain the transfer effect from slackline training seen on a task consisting to reduce as much as possible the oscillation of a swaying platform that is suddenly released (Posturomed) [10], or the capacity to rapidly counteract unexpected body sway in centre of pressure tasks [8, 11]. In the present study, the lack of improvement in a task estimating lower limb power (CMJ) could possibly explain the absence of transfer to the untrained balance tasks. In any case, the lack of generalized transfer to all of the untrained balance tasks tested indicates a possible limited transfer from slackline training to everyday life situation [13, 15]. Moreover, our result suggests that if slackline training, and by extension balance training, can ever induce general balance adaptations large enough to influence performance in all sorts of untrained tasks, a longer training duration than three months is probably necessary. It is important to point out that in the present study the training consisted only of slackline training. In the case of a multimodal training with balance training components (e.g. a training composed of strength, power, endurance and balance components), a different result in regard to generalization might occur.

Despite the three months in between the pre and post-test, the control group improved performance in many of the tested tasks. As the mere 4 trials of the pre-test were probably not enough to induce task-specific neuromuscular adaptations lasting 12 weeks and allowing a noticeable effect on performance, we suggest that factors other than skills and neuromuscular capacity might influence balance performance. For example, having already performed the task once may reduce fear of falling while performing the task, or may allow participants to use a better strategy to reach equilibrium. It must be pointed that this effect is typical in training studies with a “test-retest” approach. It has even been proposed that the presence of such effect in both the control and training group is necessary to validate a training study [21]. This time effect can be frequently observed in balance training studies (for example, see [4, 6, 16]). It is important to acknowledge the existence of such test-retest effect in balance training studies. A control group is mandatory to quantify the magnitude of such an effect and to prevent incorrect interpretation of significant improvement in performance following training. As an example, when comparing the effect of two different types of training on balance performance, an improvement in performance of untrained tasks could be observed and interpreted as transfer, indicating that both trainings can affect balance in a general way. However, the test-retest effect could also explain the better performance of both groups in the untrained tasks. Therefore, without the presence of a control group to control for such effect, no conclusive interpretation can be drawn.

Interestingly, we were not able to find a significant difference of performance for the training group between the two slackline tasks despite the fact that participants were more accustomed to the slack and properties of the slackline used during the training compared to the other tested slackline. This suggests that transfer of performance can occur after balance training if the tested untrained task is very similar to the trained task. The transfer may come from the specifically trained skills that can be directly used on this very similar task, as well as the cognitive experience acquired during the training that can help to, for example, predict the next postural perturbation to come.

It is important to point out some limitations of the present study. First, our sample size is rather small. Further, the present results are probably dependant on the overall volume and duration of the training. Increasing the training volume and/or the total training duration could potentially lead to a transfer to untrained tasks. Recently, a study showed that after 12 weeks of slackline training, young soccer players were better than a control group in untrained balance tasks, indicating a possible transfer effect induced by the training [27]. More long-term training studies are necessary to understand the generalization effect induced by balance training.

In conclusion, three months of slackline training induced only task-specific improvements in balance performance. The results of the present study underpin the results of previous short-term training studies and suggest that even several months of slackline training do not transfer to performance improvements in a broad variety of balance tasks. The present results support the concept of balance being a sum of specific skills and not a general ability. The significant test-retest effect seen in both groups emphasizes the importance of a control group to interpret the effects of balance training, especially when transfer or generalization is of interest.

## References

[1] Zech A, Hubscher M, Vogt L, Banzer W, Hansel F, Pfeifer K. Balance training for neuromuscular control and performance enhancement: a systematic review. J Athl Train. 2010;45(4):392–403. Epub 2010/07/14. 10.4085/1062-6050-45.4.392 ; PubMed Central PMCID: PMCPMC2902034. 20617915 10.4085/1062-6050-45.4.392 PMC2902034

[2] Sherrington C, Tiedemann A, Fairhall N, Close JC, Lord SR. Exercise to prevent falls in older adults: an updated meta-analysis and best practice recommendations. N S W Public Health Bull. 2011;22(3–4):78–83. Epub 2011/06/03. 10.1071/NB10056 . 21632004 10.1071/NB10056

[3] Kramer A, Dettmers C, Gruber M. Exergaming with additional postural demands improves balance and gait in patients with multiple sclerosis as much as conventional balance training and leads to high adherence to home-based balance training. Arch Phys Med Rehabil. 2014;95(10):1803–9. Epub 2014/05/16. 10.1016/j.apmr.2014.04.020 . 24823959 10.1016/j.apmr.2014.04.020

[4] Donath L, Roth R, Rueegge A, Groppa M, Zahner L, Faude O. Effects of slackline training on balance, jump performance & muscle activity in young children. Int J Sports Med. 2013;34(12):1093–8. Epub 2013/05/24. 10.1055/s-0033-1337949 . 23700328 10.1055/s-0033-1337949

[5] Donath L, Roth R, Zahner L, Faude O. Slackline training and neuromuscular performance in seniors: A randomized controlled trial. Scand J Med Sci Sports. 2016;26(3):275–83. Epub 2015/03/11. 10.1111/sms.12423 . 25756231 10.1111/sms.12423

[6] Granacher U, Iten N, Roth R, Gollhofer A. Slackline training for balance and strength promotion. Int J Sports Med. 2010;31(10):717–23. Epub 2010/08/03. 10.1055/s-0030-1261936 . 20677124 10.1055/s-0030-1261936

[7] Jager T, Kiefer J, Werner I, Federolf PA. Could Slackline Training Complement the FIFA 11+ Programme Regarding Training of Neuromuscular Control? Eur J Sport Sci. 2017;17(8):1021–8. Epub 2017/07/07. 10.1080/17461391.2017.1347204 . 28682215 10.1080/17461391.2017.1347204

[8] Keller M, Pfusterschmied J, Buchecker M, Muller E, Taube W. Improved postural control after slackline training is accompanied by reduced H-reflexes. Scand J Med Sci Sports. 2012;22(4):471–7. Epub 2011/03/10. 10.1111/j.1600-0838.2010.01268.x . 21385217 10.1111/j.1600-0838.2010.01268.x

[9] Magon S, Donath L, Gaetano L, Thoeni A, Radue EW, Faude O, et al Striatal functional connectivity changes following specific balance training in elderly people: MRI results of a randomized controlled pilot study. Gait Posture. 2016;49:334–9. Epub 2016/08/02. 10.1016/j.gaitpost.2016.07.016 . 27479219 10.1016/j.gaitpost.2016.07.016

[10] Pfusterschmied J, Stoggl T, Buchecker M, Lindinger S, Wagner H, Muller E. Effects of 4-week slackline training on lower limb joint motion and muscle activation. J Sci Med Sport. 2013;16(6):562–6. Epub 2013/01/22. 10.1016/j.jsams.2012.12.006 . 23333134 10.1016/j.jsams.2012.12.006

[11] Santos L, Fernandez-Rio J, Fernandez-Garcia B, Jakobsen MD, Gonzalez-Gomez L, Suman OE. Effects of Slackline Training on Postural Control, Jump Performance, and Myoelectrical Activity in Female Basketball Players. J Strength Cond Res. 2016;30(3):653–64. Epub 2015/09/09. 10.1519/JSC.0000000000001168 . 26349046 10.1519/JSC.0000000000001168

[12] Serrien B, Hohenauer E, Clijsen R, Taube W, Baeyens JP, Kung U. Changes in balance coordination and transfer to an unlearned balance task after slackline training: a self-organizing map analysis. Exp Brain Res. 2017;235(11):3427–36. Epub 2017/08/24. 10.1007/s00221-017-5072-7 . 28831563 10.1007/s00221-017-5072-7

[13] Thomas M, Kalicinski M. The Effects of Slackline Balance Training on Postural Control in Older Adults. J Aging Phys Act. 2016;24(3):393–8. Epub 2015/11/20. 10.1123/japa.2015-0099 . 26583953 10.1123/japa.2015-0099

[14] Volery S, Singh N, de Bruin ED, List R, Jaeggi MM, Mattli Baur B, et al Traditional balance and slackline training are associated with task-specific adaptations as assessed with sensorimotor tests. Eur J Sport Sci. 2017;17(7):838–46. Epub 2017/05/11. 10.1080/17461391.2017.1317833 . 28488937 10.1080/17461391.2017.1317833

[15] Donath L, Roth R, Zahner L, Faude O. Slackline Training (Balancing Over Narrow Nylon Ribbons) and Balance Performance: A Meta-Analytical Review. Sports Med. 2017;47(6):1075–86. Epub 2016/10/06. 10.1007/s40279-016-0631-9 . 27704483 10.1007/s40279-016-0631-9

[16] Giboin LS, Gruber M, Kramer A. Task-specificity of balance training. Hum Mov Sci. 2015;44:22–31. 10.1016/j.humov.2015.08.012 . 26298214 10.1016/j.humov.2015.08.012

[17] Kummel J, Kramer A, Giboin LS, Gruber M. Specificity of Balance Training in Healthy Individuals: A Systematic Review and Meta-Analysis. Sports Med. 2016;46(9):1261–71. 10.1007/s40279-016-0515-z . 26993132 10.1007/s40279-016-0515-z

[18] Giboin LS, Weiss B, Thomas F, Gruber M. Neuroplasticity following short-term strength training occurs at supraspinal level and is specific for the trained task. Acta Physiol (Oxf). 2018;222(4):e12998 Epub 2017/11/17. 10.1111/apha.12998 . 29144602 10.1111/apha.12998

[19] Giboin LS, Gruber M, Kramer A. Motor learning of a dynamic balance task: Influence of lower limb power and prior balance practice. J Sci Med Sport. 2018 Epub 2018/06/21. 10.1016/j.jsams.2018.05.029 . 29921504 10.1016/j.jsams.2018.05.029

[20] Hecksteden A, Faude O, Meyer T, Donath L. How to construct, conduct and analyze an exercise training study? Front Physiol. 2018;9 10.3389/fphys.2018.01007 30140237 10.3389/fphys.2018.01007 PMC6094975

[21] Green CS, Strobach T, Schubert T. On methodological standards in training and transfer experiments. Psychol Res. 2014;78(6):756–72. Epub 2013/12/19. 10.1007/s00426-013-0535-3 . 24346424 10.1007/s00426-013-0535-3

[22] Glass GV, Peckham PD, Sanders JR. Consequences of Failure to Meet Assumptions Underlying the Fixed Effects Analyses of Variance and Covariance. Review of Educational Research. 1972;42(3):237–88. 10.3102/00346543042003237

[23] Feir-Walsh BJ, Toothaker LE. An empirical comparison of the ANOVA F-test, normal scores test and Kruskal-Wallis test under violation of assumptions. Educational and Psychological Measurement. 1974;34(4):789–99. 10.1177/001316447403400406

[24] Wobbrock JO, Findlater L, Gergle D, Higgins JJ. The aligned rank transform for nonparametric factorial analyses using only anova procedures Proceedings of the SIGCHI Conference on Human Factors in Computing Systems; Vancouver, BC, Canada 1978963: ACM; 2011 p. 143–6.

[25] Zech A, Meining S, Hotting K, Liebl D, Mattes K, Hollander K. Effects of barefoot and footwear conditions on learning of a dynamic balance task: a randomized controlled study. Eur J Appl Physiol. 2018 Epub 2018/09/30. 10.1007/s00421-018-3997-6 . 30267226 10.1007/s00421-018-3997-6

[26] Gruber M, Gollhofer A. Impact of sensorimotor training on the rate of force development and neural activation. Eur J Appl Physiol. 2004;92(1–2):98–105. Epub 2004/03/17. 10.1007/s00421-004-1080-y . 15024669 10.1007/s00421-004-1080-y

[27] Trecroci A, Cavaggioni L, Lastella M, Broggi M, Perri E, Iaia FM, et al Effects of traditional balance and slackline training on physical performance and perceived enjoyment in young soccer players. Res Sports Med. 2018;26(4):450–61. Epub 2018/07/03. 10.1080/15438627.2018.1492392 . 29963921 10.1080/15438627.2018.1492392

